# The Effects of Mindfulness on Sensory Marketing: The Role of Mental Imagery Vividness and the Sensory Type Number

**DOI:** 10.3390/bs13030227

**Published:** 2023-03-06

**Authors:** Bingcan Li, Yi Jiang, Yuanzhi Wu, Lei Wang

**Affiliations:** 1 School of Psychological and Cognitive Sciences and Beijing Key Lab for Behavior and Mental Health, Peking University, Beijing 100080, China; 2 Taetea Consumer Research Center, Peking University, Beijing 100080, China; 3 Academy of Certifies Tea Master, Beijing 100871, China

**Keywords:** mindfulness, sensory marketing, purchase intention, mental imagery vividness

## Abstract

Mindfulness refers to paying attention to the present sensation, attention, and thoughts without judgment and is proven to enhance sensations. Although researchers began to investigate the role of mindfulness in consumer decision-making, few studies pay attention to the impact of mindfulness on the effect of sensory marketing. In the current study, we investigated whether and how mindfulness enhances the purchase intentions of sensory marketing products. We conducted three studies (*n* = 593) to test our hypotheses. The first study tested the correlation between trait mindfulness and the purchase intentions of sensory ads. The results showed that the level of trait mindfulness and purchase intentions were positively correlated. The second study primed the state mindfulness of participants and demonstrated that high-level state mindfulness enhanced purchase intentions, which was moderated by the number of sensory types. The third study further tested the mental imagery vividness and proved the mediating role of vividness between state mindfulness and purchase intentions. The current study shows the enhancing effect of mindfulness on purchase intentions. This effect is moderated by the number of sensory types and mediated by the vividness of mental images. Our study illustrates the critical contribution of mindfulness to promoting sensory marketing.

## 1. Introduction

Mindfulness is defined as concentrating attention consciously, in the present, and without judgment [1]. Mindfulness has a variety of effects on people’s cognition and behaviors, including physical and mental health [2,3,4], cognitive abilities [5], education and learning [6], and interpersonal relationships [7,8].

Although the concept of mindfulness has been studied in the fields of psychology for many years, its empirical research in the field of consumer psychology has only begun in recent decades [9,10,11,12,13,14]. Research has shown that mindfulness reduces overeating behaviors [15], promotes healthy eating behaviors [16], and encourages weight management [17]. In addition, from the perspective of sustainable consumption, mindfulness can enable consumers to become less materialistic [18], and can promote environmental behaviors [9]. On this basis, Milne, Villarroel Ordenes and Kaplan Milne, Villarroel Ordenes and Kaplan [11] further proposed the concept of mindful consumption, believing that mindfulness can promote conscious consumption and reduce the negative aspects of consumerism.

However, mindfulness research in consumer psychology has paid little attention to the effect of mindfulness in sensory marketing. Paying attention to one’s sensations and perceptions is one of the important components of overall mindfulness [19]. Moreover, enhanced sensations and perceptions promoted the effectiveness of sensory marketing [20]. Sensory marketing uses sensory information to attract consumers [21,22]. Effective integration of sensory information into ads can improve consumers’ pre-purchase evaluation and sensory perception of products [23]. Therefore, we proposed that mindfulness could enhance the perceptions of sensory information, which further promotes product purchase intention in sensory marketing.

In this study, we aimed to explore whether and how mindfulness enhanced sensory marketing effects. To the best of our knowledge, little is known about the effect of mindfulness in sensory marketing. Specifically, we proposed that high-level mindfulness made consumers more sensitive to sensory information in sensory ads, which leads to a positive impact on product purchase intention. As demonstrated in previous studies, mindfulness can be conceptualized as either a stable trait or a state that changes with the situation [24]. Therefore, the current study aims to show that mindfulness both as a trait and a state, facilitates purchase intentions for products through multisensory ads. In the current research, we conducted three studies to investigate the impact of mindfulness on the effect of sensory marketing and the effect of the number of sensory types and mental imagery vividness.

## 2. Theoretical Background

### 2.1. Mindfulness

Mindfulness is defined by two major perspectives. From the traditional Buddhist perspective, mindfulness is defined as “concentrating attention in a specific way, which is paying attention consciously, in the present, and without judgment” [1]. From the cognitive perspective, mindfulness is defined as a heightened state of awareness of external stimuli [25]. Although these two definitions define mindfulness from different perspectives of non-judgment and engagement, they both focus on the perception and awareness of external stimuli.

Research on the relationship between mindfulness and cognition, personality, and social psychology constructs shows that mindfulness should be used more as a cognitive style than just a specific cognitive ability or personality trait [24,26]. Therefore, mindfulness should be regarded as a construct of both personality traits and cognitive ability. It can be used as both a state variable and a trait variable.

In consumer psychology, many studies on mindfulness in marketing explore the role of mindfulness from the perspective of awareness. For example, previous studies showed that mindfulness increased conscious consumption [9,27,28]. Mindfulness helps consumers to maintain an awareness of the consumption process, promote sustainable consumption, and optimize the consumption experience [11]. Additionally, from the perspective of food consumption, mindfulness also helps to increase consumers’ awareness of the eating process, thereby reducing unconscious eating behaviors [15] and promoting healthy eating behaviors [16].

However, although mindfulness also enhances sensations and perceptions [3], the research on the sensory-enhancing role of mindfulness in consumption is still limited. For example, Van De Veer, Van Herpen, and Van Trijp Van De Veer, Van Herpen and Van Trijp [14] show that mindful attention directed to the body, as opposed to the environment, leads to an awareness of satiety cues and, accordingly, an adjustment of further food intake. Therefore, our aim was to explore whether mindfulness could promote the perceptions of sensory information in marketing.

### 2.2. Mindfulness and Sensory Marketing

Studies show that mindfulness can play an role in enhancing sensation and attention [5]. This is because individuals with high levels of mindfulness (1) are more open to new information, pay more attention to details [29], have higher attention and concentration capabilities, and therefore develop stronger observation capabilities [30]; (2) have more receptive attention and awareness of current happenings [3], and higher levels of engagement [31]; (3) have greater sensitivity and interest in their surroundings [32]; (4) have stronger cognitive categorization capabilities, and can pay attention to more perspectives when solving problems [33]; and (5) can react more flexibly to contextual cues [34].

Moreover, enhanced sensory awareness in the mindful state [3] is especially necessary for sensory marketing. Sensory ads provide consumers with direct or indirect sensory clues to attract consumers’ senses [21]. Direct sensory marketing means providing consumers with direct sensory products to convey sensory clues, for example, using scented paper strips in magazine advertisements for perfume to provide olfactory stimulation [35]. Indirect sensory marketing means that the consumers’ imagination is activated through words or images to convey sensory clues, such as depicting multisensory information through advertising copy [36]. However, sensory information in marketing does not necessarily lead to susceptibility, and the effect of sensory information on attracting consumers is affected by situational and personal factors [20]. Therefore, mindfulness helps consumers to enhance their sensations towards sensory clues, which promotes the effects of sensory marketing.

In the current study, we aim to explore the role of mindfulness in enhancing the effect of sensory marketing. Since mindfulness is both a trait and a state variable, the current study explores the relationship between mindfulness and the effects of sensory advertising from the perspectives of both trait and state mindfulness. Consumers with a high level of trait mindfulness have a stronger perception of the sensory information in the advertisement copy [19], so they can vividly imagine the sensory attributes of the products in the advertisement copy. Previous studies have shown that the perception of sensory information regarding products in sensory marketing can enhance product purchase intention [22]. Accordingly, we hypothesize:

**Hypothesis 1** **(H1).** *The level of trait mindfulness is positively correlated with purchase intentions in sensory marketing.*

Similarly, when the mindfulness state of consumers is primed, consumers’ attention and consciousness will be more focused, and their sensory ability will also be enhanced [14]. Therefore, the consumers primed with mindfulness have a stronger perception of the multisensory information in the advertisement. In this case, priming the mindfulness state of the consumers (compared with the control group) can lead to higher purchase intentions for the products in the sensory ads. Accordingly, we hypothesize:

**Hypothesis 2** **(H2).** *Compared with the control condition, the priming of state mindfulness enhances purchase intentions in sensory marketing.*

### 2.3. The Moderating Role of the Number of Sensory Types in Sensory Marketing

In sensory marketing, compared to single-sense advertising copy, increasing the number of sensory types can increase consumers’ purchasing intentions [23]. Food ads, for example, descriptions of other sensory types, in addition to taste, can complement the consumer’s overall perception of the food. There have been some studies that have explored the of the other senses combined with taste [37,38]. For example, smell affects the taste of food before and after it enters the mouth [39] and plays an essential role in taste perception [40]. In addition, the visual appearance of food also contributes to the expectations and perception of taste [37]. The sound that food makes when bitten also plays a key role in the taste perception of certain foods, affecting perceived freshness and perceived quality [38]. Finally, tactile perceptions such as texture and mouthfeel of food [41] also influence taste perception.

According to the characteristics of mindfulness, consumers with a high level of mindfulness have higher motivation, sensation, and competence in their information processing [30]. They have a higher perception of sensory information and can pay more attention to sensory product characteristics. Therefore, compared with fewer numbers of sensory types, multiple sensory information can provide consumers with more sensory clues, thus helping consumers with a high level of mindfulness to have a stronger perception of sensory information and a higher purchasing intention. Therefore, the number of senses can strengthen the influence of mindfulness on purchase intention in multisensory marketing. Based on this, we propose:

**Hypothesis 3** **(H3).** *The number of sensory types plays a moderating role in mindfulness and purchase intentions, such that with the increase of the number of senses, mindfulness plays a stronger role in promoting purchase intention.*

### 2.4. The Mediating Role of the Mental Imagery Vividness

Mental imagery vividness refers to the clarity of the imagined scene [42]. Many researchers believe that vividness is the most important aspect of mental imagery [43]. Previous studies demonstrated that the level of the vividness of individual mental images is positively correlated with the level of mindfulness, both for trait mindfulness and state mindfulness [44,45]. For example, Bedford Bedford [44] found that in the process of meditation practice, differences in individual mindfulness levels can predict differences in the vividness of their mental images. Kharlas and Frewen Kharlas and Frewen [45] further proved that individuals with higher levels of trait mindfulness can visualize stimuli in multiple sensory modes, whether during or outside of meditation practice events. Therefore, it can be speculated that when faced with sensory information, individuals with high levels of mindfulness may produce more vivid mental images than those with low levels of mindfulness.

Moreover, mental imagery vividness is considered to have a positive impact on consumers’ judgments [46,47,48]. Vivid, self-related, and reasonable stimuli will directly and positively affect people’s attitudes towards advertising and marketing by enhancing the vividness of mental images [46]. Additionally, guiding participants to imagine the product experience may have a negative impact on product preference when the participant’s mental imagery ability is low or the product is presented in a vague way [47]. Previous studies have shown that the vividness of mental imagery can play a mediating role between stimuli (e.g., specific words) and consumers’ attitudes and cognitive responses [49,50].

In advertising copy, the use of multisensory information facilitates the creation of a vivid mental image, which further serves to enhance purchase intentions [21]. Although other senses, such as smell and taste, cannot be directly triggered in online communications, marketers can indirectly trigger mental images of these senses through text or images, thereby promoting product experience based on these senses. Advertising copy plays an important role in conveying multisensory information about products [36]. Specific and clear text descriptions are called “concrete stimuli”, which can make people’s mental images more vivid [49,51,52]. Studies have shown that effectively integrating multisensory information into online advertising copy can improve consumers’ pre-purchase evaluation of products [23]. For example, when promoting the newly launched chocolate Indian Tea Mandarin Latte, Starbucks used consumers’ senses to create an attractive consumption scene by describing its taste, aroma, and appearance.

As mentioned above, mindfulness can promote mental imagery vividness, and mental imagery vividness can further promote purchase intentions. We, therefore, hypothesize that mindfulness can promote purchase intentions by increasing the vividness of consumers’ mental imagery in response to multisensory information. Accordingly, we propose:

**Hypothesis 4** **(H4).** *The vividness of mental imagery plays a mediating role in the effects of mindfulness on product purchase intentions.*

### 2.5. Overview of the Present Studies

**Figure 1:**
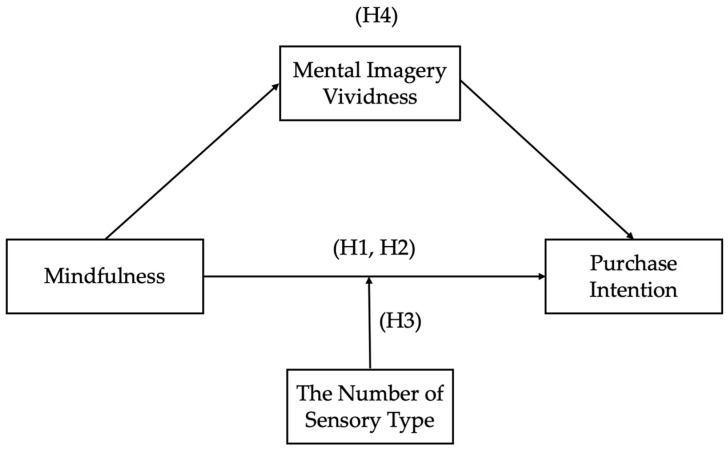
A conceptual model of the hypotheses.

The concept model is depicted in Figure 1.

We tested our hypotheses across four studies. In Study 1, we investigated the correlation between trait mindfulness and purchase intentions in sensory ads. Study 2 used audio to prime state mindfulness to explore the influence of mindfulness on purchase intentions. In addition, we further tested the moderating effect of the number of sensory types. Study 3 further explored the mediating effect of mental imagery vividness. In order to ensure homogeneity among the participants in the three studies, participants were all selected from the same online sample pool and randomly assigned to Study 1, 2, or 3. The sample pool was chosen from the Credamo platform (https://www.credamo.com (accessed on 1 March 2021)), a commercial Chinese company that provides online survey services.

## 3. Study 1

Study 1 tested the first hypothesis that trait mindfulness is positively correlated with purchase intentions for the product in sensory ads. In the study, we measured the trait mindfulness variable of the participants. Specifically, we used the Mindful Attention Awareness Scale developed by Brown and Ryan Brown and Ryan [3] to measure the trait level of individuals’ awareness and attention to the present. Next, we asked the participants to read the sensory advertising copy about dried mango, and then measured their purchase intentions with regard to dried mango.

### 3.1. Participants

Participants were recruited from the Credamo platform. In total, 150 participants completed the survey. Each participant received one yuan (RMB) in exchange for participating. Eight of the subjects reported that they usually did not eat mangoes, so they may not be able to imagine the description in the advertisement copy. Therefore, the data of these eight subjects were eliminated. One hundred and forty-two valid participants were finally included in the subsequent analysis. Among them, 62 were males (43.7%), and 80 were females (56.3%). The ages of the subjects were 18–47 years old, with an average age of 28.61 years (*SD* = 5.31).

### 3.2. Procedure

In this study, participants were asked to complete a series of questions online. First, participants were asked to answer questions in the Mindful Attention Awareness Scale (MAAS; Brown & Ryan, 2003), which was used to measure mindfulness traits by assessing one’s attention and awareness in the daily life (e.g., “I find it difficult to focus on what is happening at present”; 15 items; α = 0.91). Participants rated the extent to which they agree with each item (1 = “very agree”; 5 = “very disagree”). To score the MAAS scale, we computed the mean of the 15 items.

Afterwards, participants were asked to read advertising copy introducing freeze-dried mangoes. We set up three sets of freeze-dried mango advertising copy that included one, three, or five senses for the subjects to present at random. Advertising copy was modified from that designed by Elder & Krishna (2010). The advertising copy read as follows:

(1) Single sense (taste):

*“Imagine the long-awaited taste of our natural freeze-dried mango. Open it and you can taste the taste from tropical orchards at home. Each freeze-dried mango can make you eat fresh and full pulp. It has a sweet and rich taste. When you are tasting it, the sweet taste jumps on the tip of your tongue like a beautiful dance. Our natural freeze-dried mango is the perfect choice for your snack”;*

(2) Three senses (taste, smell, and vision):

*“Imagine the long-awaited taste (taste) of our natural freeze-dried mango. Open it and you can smell the smell from tropical orchards (smell) at home. Each freeze-dried mango allows you to see the fresh and full flesh (vision). It has a sweet and rich taste. When you are tasting it, the sweet taste jumps on the tip of your tongue like a beautiful dance. Our natural freeze-dried mango is the perfect choice for your snack”;*

(3) All five senses (taste, smell, vision, hearing, and touch):

*“Imagine the long-awaited taste (taste) of our natural freeze-dried mango. Open it and you can smell the smell from tropical orchards (smell) at home. Each freeze-dried mango allows you to see the fresh and full flesh (vision). It has a crisp texture (touch). When you bite it, the sound of clicking is like wonderful music around your ear (hearing). Our natural freeze-dried mango is the perfect choice for your snack”.*

It is worth noting that we made the three sets of advertising copy the same in two dimensions of “information richness” and “overall evaluation” with the same word counts, but with different numbers of sensory types mentioned. To achieve this goal, we used a group of unrelated subjects to pre-test three groups of advertising copy (*n* = 90). In the pre-test, participants scored 7 points on the richness of information provided by the advertising copy and the overall evaluation of the advertising copy (three items, α = 0.74) [23,53]. The results found that the three sets of materials showed no difference in the two dimensions of information richness (*M*_1_ = 6.00, *M*_3_ = 6.00, *M*_5_ = 6.03) and overall evaluation [(*M*_1_ = 17.33, *M*_3_ = 18.03, *M*_5_ = 17.80) (*F_richness_* (2, 87) = 0.016, *p* = 0.984; *F_overall evaluation_* (2, 87) = 0.854, *p* = 0.429). Paired comparisons showed that there was no significant difference in the scores of any two groups in the two dimensions (*ps* > 0.05).

Next, the subjects were required to answer a question to measure their purchase intentions [54] on a 9-point scale (1 = “very reluctant to buy”, 9 = “very willing to buy”). We used a 9-step Likert scale. We used a 9-point scale to test the purchase intentions for products, which was adopted from the 9-point Hedonic scale [55]. Consumers rated their purchase intentions using the scale, which was presented as follows: “[1] 2 3 4 [5] 6 7 8 [9]”. The extremes, “1” and “9”, represented “extremely reluctant” or “extremely willing”, respectively, and “5” represented a neutral attitude. While “2” represented a strong reluctance, “3” represented a moderate level of reluctance, and “4” represented a slight reluctance. Similarly, “8” represented a strong willingness, “7” represented a moderate level of willingness, and “6” represented a slight willingness. To exclude the potential influence of the subject’s personal preference for the product itself, we asked the subjects whether they usually ate mangoes. Finally, the demographic information of the subjects was collected, including gender, age, and education level.

### 3.3. Results and Discussion

To examine the effects of trait mindfulness on purchase intention, a linear regression analysis was performed. Trait mindfulness was entered as the independent variable and purchase intention was entered as the dependent variable. The gender and age of participants were included as covariates. The results show the significant effect of mindfulness on purchase intentions (*Beta* = 0.30, *t* (138) = 3.76, *p* = 0.000). Additionally, the correlation between mindfulness and purchase intentions was also significant (*r*^2^ = 0.31, *p* < 0.001). These results indicated that a higher score on trait mindfulness was associated with higher purchase intention.

The results of Study 1 supported H1. The trait mindfulness level was significantly correlated with purchase intentions related to products in sensory ads. This result showed that those participants who have a high level of trait mindfulness in daily life are more willing to buy products that use sensory advertising. However, this study only obtained correlation evidence through measurement. The causal relationship between mindfulness and sensory marketing effects remains unclear. Therefore, in the following study, we explored whether state-induced mindfulness affects the purchase intention of products in sensory ads by priming mindfulness through experiments.

## 4. Study 2

In Study 1, we used the survey method to investigate the correlation between mindfulness and purchase intentions with regard to products in sensory ads. However, the positive correlation could not determine the direction of causality. Therefore, in Study 2 and Study 3, we used experimental designs to investigate the causal relationship between mindfulness and purchase intentions. Specifically, we randomly assigned participants to either a mindfulness group or a control group to determine the effect of mindfulness on purchase intentions.

The purpose of Study 2 is to explore the influence of mindfulness on purchase intentions by priming state mindfulness (H2). In addition, Study 2 aims to evaluate the moderating effect of the number of sensory types on the relationship between mindfulness and product purchase intentions (H3).

### 4.1. Participants

In the current study, participants were recruited from the Credamo platform. In total, 222 participants completed the survey. Two of the participants failed the attention test, and six participants failed to complete the mindfulness priming task. Therefore, the data of these eight participants were eliminated. Finally, there were 214 valid participants. Among them, 108 were males (50.5%), and 106 were females (49.5%). The ages of the participants were 19–47 years old, with an average age of 28.37 years (*SD* = 5.24).

### 4.2. Research Design

This study adopted a 2 (mindfulness priming: mindfulness group vs. control group) × 3 (the number of sensory types: one vs. three vs. five) between-subjects research design to examine the influence of mindfulness and to verify the moderating effect of the number of sensory types. The participants were randomly assigned to one of the above six situations.

### 4.3. Procedure

First, participants were randomly assigned to either the mindfulness group or the control group. They were asked to finish the corresponding audio listening task. The material in the mindfulness group is a mindfulness practice audio, and the material in the control group is a neutral audio introduction to natural science phenomena.

In the mindfulness condition, participants heard the mindfulness practice audio, which is 3.5 min long and read by male professionals, which was adapted from Van De Veer, Van Herpen, and Van Trijp Van De Veer, Van Herpen and Van Trijp [14]. The mindfulness practice audio asked participants to pay attention to their current thinking, breathing, and physical feelings. Specifically, participants were first asked to choose an appropriate posture for meditation practice. They were instructed to pay attention to their current feelings and emotions (e.g., “*please pay attention to any emotional discomfort or unpleasant feelings and admit their existence*”). Next, they were asked to pay attention to their current breathing (e.g., “*focus your attention on the physiological feeling of breathing and feel the feeling of breathing in the abdomen at a close distance*”) and follow their breathing for physical relaxation (e.g., “*in addition to the feeling of breathing, please feel the feeling of the whole body, including your body and face*”). In the control condition, participants heard audio that was a short essay on the rain and the weather. The readers of both audios were male, and the voice and intonation were controlled to be consistent to ensure that irrelevant factors did not have an impact on the priming effect. To ensure the validity of the audio priming, the instructions required the participants to wear headphones and be in a quiet environment to complete the audio task.

After listening to the audio, participants were required to answer five questions to measure their state mindfulness level as a manipulation check. The measurement was adapted from Van De Veer, Van Herpen, and Van Trijp Van De Veer, Van Herpen and Van Trijp [14] (e.g., “*I was very clearly aware of my own physical processes.*”) (α = 0.81). The questions are scored from 1 (completely inconsistent) to 7 (completely consistent). To score the mindfulness, we computed the mean of the 5 items.

Afterwards, in accordance with Study 1, participants were asked to read advertising copy, including one, three, or five senses at random. Then they were also required to answer a question to measure their purchase intentions on a 9-point Likert scale (1 = “very reluctant to buy”, 9 = “very willing to buy”). To exclude their personal preference for the product itself, we asked the subjects to rate their preference for mangoes. Finally, the demographic information of the participants was collected, including gender, age, education level, and job type.

### 4.4. Statistical Analysis

We conducted an ANOVA to test the impact of mindfulness and the number of sensory types on purchase intentions (H2 and H3), with condition (mindfulness vs. control) and the number of sensory types (one vs. three vs. five) as independent variables, and purchase intentions as dependent variables. We reported the main and interactional effects of the ANOVA.

### 4.5. Results and Discussion

The independent t-test on mindfulness showed that the scores of the state mindfulness level of the participants in the mindfulness group (*M_mindfulness_* = 5.81, *SD_mindfulness_* = 0.61) were significantly higher than those of the control group (*M_control_* = 5.38, *SD_control_* = 0.93), *t* (212) = 4.01, *p* <.001, *d* = 0.55, indicating that the priming of mindfulness is effective. In addition, no significant correlation between mango preference and purchase intentions was found. See Table 1 for detailed information.

To test our hypothesis, a 2 (condition: mindfulness group vs. control group) × 3 (the number of sensory types: one vs. three vs. five) ANOVA was conducted.

First, the results showed that the effect of mindfulness was significant [*F* (1, 208) = 65.25, *p* = 0.000, η_p_^2^ = 0.24]. The purchase intentions of the mindfulness group (*M_mindfulness_* = 7.80, *SD_mindfulnesse_* = 0.70) were higher than those of the control group (*M_control_* = 7.06, *SD_control_* = 0.79), verifying the enhancing effect of mindfulness. This result supported Hypothesis 2.

Second, the results showed that the effect of the number of sensory types was also significant, *F* (2, 208) = 22.92, *p* = 0.000, η_p_^2^ = 0.18. Paired comparisons revealed that the five-sensory group (*M*_5_ = 7.81, *SD* = 0.83, *p* < 0.001, *d* = 0.97) and the three-sensory group (*M*_3_ = 7.41, *SD* = 0.77, *p* = 0.006, *d* = 0.47) have significantly higher product purchase intentions than the one-sensory ad condition (*M*_1_ = 7.06, *SD* = 0.71). In addition, the purchase intentions in the five-sensory ad condition were also significantly higher than those in the three-sensory ad condition (*p* = 0.003, *d* = 0.50).

Third, the results showed that there was a significant interaction between the number of sensory types and mindfulness, *F* (2, 208) = 5.92, *p* = 0.003, η_p_^2^ = 0.054. Paired comparison showed that in the one-sensory ad condition, the difference of purchase intentions between the mindfulness and control groups was significant [*t* (70) = 2.78, *p* = 0.07]. In the three-sensory ad condition, the difference of purchase intentions between the mindfulness group and the control group was also significant [*t* (68) = 3.45, *p* = 0.001]. In the five-sensory ad condition, the difference of purchase intentions between the mindfulness group and the control group was more significant [*t* (70) = 8.32, *p* = 0.000].

**Figure 2:**
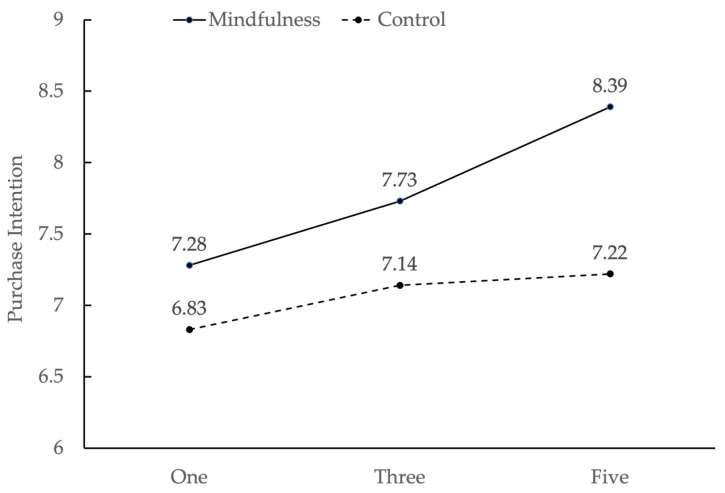
The interaction of purchase intentions between the number of sensory types and mindfulness in Study 2.

These results showed that the number of sensory types played a moderating role in the relationship between mindfulness and purchase intentions, which is in line with Hypothesis 3. The interaction diagram is shown in Figure 2.

The results of Study 2 support both Hypotheses 2 and 3. Compared to the control group, the purchase intention was higher in the mindfulness group, confirming the enhancing effects of mindfulness. Moreover, when the number of sensory types included in the advertising copy increased, the effects of mindfulness on purchase intentions increased accordingly. In the following study, we will explore the mechanism of the relationship between mindfulness and purchase intentions.

## 5. Study 3

The purpose of Study 3 is to repeat the findings of Study 2 and further explore the mediating role of mental imagery vividness. Specifically, Study 3 investigated whether the process in which state mindfulness affects purchase intentions is mediated by the vividness of mental images (H4).

### 5.1. Participants

In the current study, 259 participants were recruited from the Credamo platform, completing the survey online. Ten of the subjects failed the attention test, and twelve subjects failed to complete the audio listening task. Therefore, there were 237 valid participants. Among them, 109 were males (46%), and 128 were females (54%). The ages of the participants were 19–47 years old, with an average age of 28.40 years (*SD* = 5.28).

### 5.2. Research Design

The current study adopted a 2 (mindfulness priming: mindfulness group vs. control group) × 3 (the number of sensory types: one vs. three vs. five) between-subjects design. Both mindfulness and independent variables were manipulated in the same way as in Study 2. To explore the mediating mechanism, the current study added the measure of the vividness of mental images [50].

### 5.3. Procedure

The first part was the priming of mindfulness. The same audio introductions were used as those described in Study 2 as the priming/control materials. Participants were randomly assigned to one of the two groups (mindfulness group/control group) and were asked to finish the same tasks of listening to the audio advertisements.

After listening to the audio and rating their purchase intentions about the advertisement copy, participants were asked to answer four questions that measure the vividness of mental images on a 7-point scale [56] (e.g., “*How vivid do you think the product description in the ads copy is?*”, α = 0.83).

Finally, the demographic information and mango preference of the subjects were collected.

### 5.4. Statistical Analysis

First, we conducted an ANOVA to test the impact of mindfulness and the number of sensory types on purchase intentions (H2 and H3), with condition (mindfulness vs. control) and the number of sensory types (one vs. three vs. five) as independent variables, and purchase intentions as dependent variables.

Next, we conducted an ANOVA to test the impact of mindfulness and the number of sensory types on mental imagery vividness, with condition (mindfulness vs. control) and the number of sensory types (one vs. three vs. five) as independent variables, and mental imagery vividness as the dependent variable.

Finally, we used Hayes’ [57] bootstrapping procedure (10,000 iterations) to test the mediating role that vividness has in the effect of mindfulness on product purchase intentions (H4).

### 5.5. Results and Discussion

#### 5.5.1. The Manipulation Checks of Mindfulness

A paired sample t-test proved that the scores of the state mindfulness level of the participants in the mindfulness group (*M* = 5.83, *SD* = 0.78) were significantly higher than those of the control group (*M* = 5.40, *SD* = 0.96), *t* (235) = 3.68, *p* < 0.001, *d* = 0.61. The result of the manipulation test shows that it is effective for the priming of mindfulness.

#### 5.5.2. The Influence of Mindfulness and the Number of Sensory Types on Purchase Intentions

A 2 (mindfulness priming: mindfulness group vs. control group) * 3 (the number of sensory types: one vs. three vs. five) ANOVA was conducted with the purchase intentions as the dependent variable.

First, the results showed that the effect of mindfulness was significant [*F* (1, 231) = 80.65, *p* = 0.000, η_p_^2^ = 0.26]. The purchase intentions of the mindfulness group were higher than that of the control group (*M_mindfulness_* = 7.76, *SD_mindfulness_* = 0.78; *M_control_* = 7.05, *SD*_control_ = 0.62).

Second, the results showed that the effect of the number of sensory types was also significant, *F* (2, 231) = 44.52, *p* = 0.000, η_p_^2^ = 0.28. Post hoc analysis revealed that the five-sensory ad condition (*M*_5_ = 7.79, *SD* = 0.81, *p* < 0.001, *d* = 1.12) and the three-sensory ad condition (*M*_3_ = 7.35, *SD* = 0.65, *p* < 0.001, *d* = 0.51) had significantly higher purchase intentions than the one-sensory ad condition (*M*_1_ = 6.93, *SD* = 0.59). In addition, purchase intentions in the five-sensory ad condition were also significantly higher than those in the three-sensory ad condition (*p* < 0.001, *d* = 0.69).

Third, the results showed that there was a significant interaction between mindfulness and the number of sensory types, *F* (2, 231) = 13.38, *p* = 0.000, η_p_^2^ = 0.10. Paired comparison showed that in the one-sensory ad condition, the difference of purchase intentions between the mindfulness and the control group was significant [*t* (71) = 2.19, *p* = 0.03]. In the three-sensory ad condition, the difference of purchase intentions between the mindfulness group and the control group was also significant [*t* (80) = 3.93, *p* = 0.000]. In the five-sensory ad condition, the difference of purchase intentions between the mindfulness group and the control group was more significant [*t*(80) = 9.83, *p* = 0.000]. These results also showed that with the increase of the number of senses, the enhancing effect of mindfulness became stronger. These results were in consistent with those in Study 1, supporting H2 and H3.

#### 5.5.3. The Influence of Mindfulness and the Number of Sensory Types on the Mental Imagery Vividness

To test our hypothesis, a 2 (mindfulness priming: mindfulness group vs. control group) × 3 (the number of sensory types: one vs. three vs. five) ANOVA was conducted with mental image vividness as the dependent variable.

First, the results showed that the effect of mindfulness was significant [*F* (1, 231) = 106.65, *p* = 0.000, η_p_^2^ = 0.32]. The mental imagery vividness of the mindfulness group was higher than that of the control group (*M_mindfulness_* = 6.18, *SD_mindfulness_* = 0.38; *M_control_* = 5.74, *SD_control_* = 0.38), verifying the enhancing effect of mindfulness on vividness.

Second, the results showed that the effect of the number of sensory types was also significant, *F* (2, 231) = 49.40, *p* = 0.000, η_p_^2^ = 0.30. Post hoc analysis revealed that the five-sensory ad condition (*M*_5_ = 6.18, *SD* = 0.47, *p* < 0.001, *d* = 1.38) and the three-sensory ad condition (*M*_3_ = 5.93, *SD* = 0.38, *p* < 0.001, *d* = 0.84) had significantly higher mental imagery vividness than the one-sensory ad condition (*M*_1_ = 5.67, *SD* = 0.30). In addition, the mental imagery vividness of the five-sensory ad condition was also significantly higher than that of the three-sensory ad condition (*p* < 0.001, *d* = 0.59).

**Figure 3:**
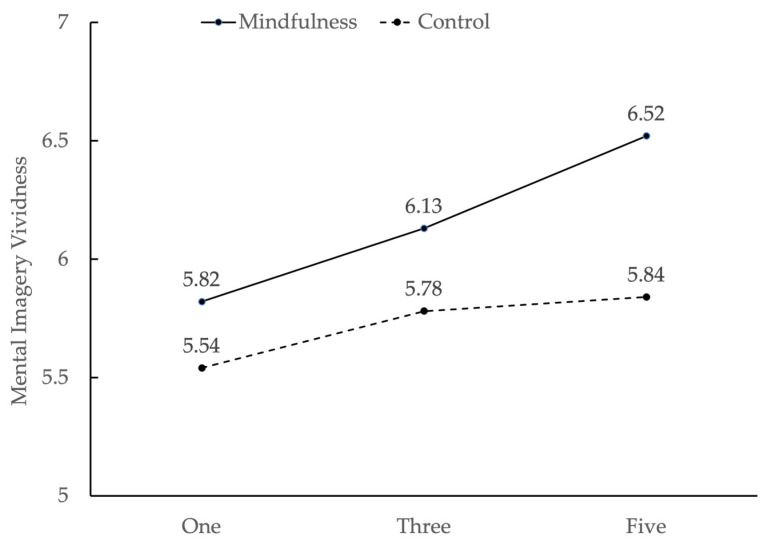
The interaction of mental imagery vividness between the number of sensory types and mindfulness in Study 3.

Third, the results showed that there was a significant interaction between mindfulness and the number of sensory types, *F* (2, 231) = 7.98, *p* = 0.000, η_p_^2^ = 0.065. In the control group, participants in the five-sensory ad condition (*M*_5_ = 5.86, *SD* = 0.40) and the three-sensory ad condition (*M*_3_ = 5.78, *SD* = 0.36) had significantly higher mental imagery vividness than those in the one-sensory ad condition (*M*_1_ = 5.54, *SD* = 0.31, *p_5v1_* = 0.000; *p_3v1_* = 0.003). However, the mental imagery vividness of the five-sensory ad condition was not significantly higher than that of the three-sensory ad condition (*p* > 0.05). In the mindfulness groups, participants in the five-sensory ad condition (*M*_5_ = 6.52, *SD* = 0.24) and the three-sensory ad condition (*M*_3_ = 6.13, *SD* = 0.31) had significantly higher mental imagery vividness than those in the one-sensory ad condition (*M*_1_ = 5.82, *SD* = 0.20, *p_5v1_* = 0.000; *p_3v1_* = 0.000). Additionally, the mental imagery vividness in the five-sensory ad condition was significantly higher than that in the three-sensory ad condition (*p* = 0.000). These results indicated that in the control group, the vividness did not increase significantly as the number of sensory types increased. However, in the mindfulness group, the vividness increased with the number of sensory types, suggesting that mindfulness could enhance the vividness of multisensory advertising copy (see Figure 3).

#### 5.5.4. The Mediating Effect of Mental Imagery Vividness on the Relationship between Mindfulness and Purchase Intentions

We estimated the mediation model using mindfulness as the independent variable (1-mindfulness group; 0-control group), the mental imagery vividness as the mediator variable, and purchase intentions as the dependent variables. The bootstrapping technique with 95% confidence intervals and 10,000 samples was conducted to test for mediation (Hayes, 2017, Hayes’ Model 4). The results revealed that the indirect effect of mindfulness on purchase intentions via the mental imagery vividness was significant, B = 0.23, [LLCI: 0.1223, ULCI: 0.3558]. This finding supported the mediating effect of mental imagery vividness on the relationship between mindfulness and purchase intentions, supporting H4.

The results of Study 3 replicated the findings of Study 2, supporting H2 and H3. Additionally, Study 3 showed that participants in the mindfulness group perceived higher mental imagery vividness in relation to the product than those in the control group. In addition, mental imagery vividness played a partial mediating role in the influence of mindfulness on purchase intentions, supporting H4.

## 6. General Discussion

### 6.1. Summary of the Findings

This research links mindfulness with multisensory marketing and uses empirical research methods for the first time in order to demonstrate that mindfulness can promote the effects of sensory information in advertisements on purchase intentions. In addition, it also shows the mediating effect of the vividness of mental images and the moderating role of the number of sensory types.

In the survey study (Study 1), participants with higher scores on the mindfulness traits scale have higher levels of mindfulness. Conversely, participants with lower scores have lower levels of mindfulness. In Study 1, the mean rating exceeding the average value of *M* = 3.4 represented a high level of mindfulness. The mean rating below the average of *M* = 3.4 represented a low level of mindfulness. In the experimental studies (Study 2 and 3), the level of mindfulness was manipulated through an experimental manipulation of mindfulness. Participants in the mindfulness group had a higher level of mindfulness due to completing the mindfulness priming task. The control group had a lower level of mindfulness because they completed an irrelevant task. In Study 2 and Study 3, the mean rating exceeding the average value of *M* = 5.6 represented a high level of mindfulness. The mean rating below the average of *M* = 5.6 represents a low level of mindfulness.

First, these results show that compared to individuals with low levels of mindfulness, people’s purchase intentions were significantly higher for individuals with high levels of mindfulness. We confirmed the positive correlation between mindfulness and purchase intentions from both trait mindfulness and state mindfulness. Previous studies found that individuals with high levels of mindfulness have a stronger perception of the sensory information in the ad copy [19], and their attention will be more focused on details [25]. Therefore, consumers with a high level of mindfulness have higher levels of engagement and sensory ability in their decision-making for sensory ads [2,31], which makes them pay more attention to multiple sensory information in advertisement copy and further lead to a positive influence on purchase intentions.

In addition, the results of the current research show that mental imagery vividness has a mediating effect on the relationship between mindfulness and purchase intentions. This result is consistent with the findings of previous research on mental imagery vividness. It has been found that mental imagery vividness has a positive impact on consumers’ judgments and intentions [46]. Burns, Biswas, and Babin Burns, Biswas and Babin [49] demonstrated that mental imagery vividness can play a mediating role between the stimulus and the consumer’s attitude and cognitive response. Individuals with high levels of mindfulness also have a higher working memory capacity than individuals with low levels of mindfulness [5]. Therefore, when dealing with multisensory information, individuals with higher levels of mindfulness will produce more vivid mental images than individuals with lower levels of mindfulness due to their richer working memory resources.

In addition, compared with those in the control group, the purchase intentions in the mindfulness group increased with the increase in the number of sensory types. In other words, the positive influence of multiple pieces of sensory of information on purchase intentions is enhanced by mindfulness. This is probably because when the number of sensory types increases to a certain degree, individuals with low levels of mindfulness find it difficult to cope with increasing sensory information, so the positive effect of multisensory information on product evaluation is weakened [23]. However, mindfulness can help consumers enhance their perception of multisensory information in sensory advertising, so that the positive effect of multisensory information on product evaluation can be maintained.

### 6.2. Theoretical Implications

The results of current research extend prior theorizing in several important ways. First, this research contributes to the literature on mindfulness, explaining that mindfulness enhances the purchase intentions for products in sensory advertisements. Previous studies have found that mindfulness had a positive impact on consumers’ product evaluation and decision-making processes [12]. Previous studies also found that mindfulness could enhance attention, thus increasing the perception of the physiological state after food consumption [14]. We provide evidence that the sensory enhancing effect of mindfulness can also increase mental imagery vividness, and thus enhance purchase intentions for products with sensory marketing.

Second, our research contributes to the research on the antecedents of sensory marketing effectiveness by explaining the role of mindfulness in enhancing purchase intentions for products. Our findings reveal the influential role of mindfulness on sensory marketing, both for trait mindfulness and state mindfulness. Previous studies have shown that in the sensory marketing of food, the advertising effect of mentioning multiple senses was better than that of only mentioning taste, by improving the overall taste perception of consumers [23]. Our research demonstrates that the perception of sensory information in advertisements can also be affected by mindfulness, thus broadening the theories of sensory marketing from the perspective of consumers.

Third, our research contributes to the literature on mental imagery vividness in sensory consumption by exploring the mediating role of vividness. Previous studies have found that mindfulness and the vividness of mental images still correlate outside of meditation practice [45] and the vividness of mental images has a positive influence on consumers’ judgment and intention [46]. Consistent with this line of research, our research examines the mediating role of vividness and demonstrates that individuals with high mindfulness will produce more vivid mental images and further enhance purchase intentions in sensory ads.

### 6.3. Practical Implications

The findings of the current research also have practical implications for consumer psychology.

In the results of Study 2 (Figure 2), it can be seen that in the control group (i.e., the group without mindfulness priming), the effect of sensory marketing increased with the increase of the number of sensory types, but the increase was very limited (6.83 to 7.22). However, when the participants were primed by the midfulness task, the purchase intentions increased significantly (7.28 to 8.39). The same results were replicated in Study 3. These results demonstrate that mindfulness plays a positive role in promoting the effect of sensory marketing, especially when the number of sensory types is relatively large. This provides a research basis for the application of mindfulness in the field of sensory marketing in the future. Therefore, our research will bring awareness to marketers about the importance of fostering individual mindfulness to influence the effectiveness of sensory marketing. For example, when conducting sensory marketing, marketers can choose sound or guidance to induce consumers’ high mindfulness in advertisements or find a time and a place with a high mindfulness level for marketing, which can achieve better marketing results. Specifically, they can adopt meditation background music in sensory ads that can improve the level of state mindfulness. As technology-enabled sensory marketing is developing [58], the current study contributes to sensory marketing by exploring the use of mindfulness in sensory advertising on digital platforms.

### 6.4. Limitations and Future Directions

Even though the current research offers important theoretical and practical implications, there are also areas for improvement in this research. First, the manipulation of consumers’ mindfulness levels in this study is primed through audio or written materials. To make the research results more generalizable in practical applications, future research can consider incorporating mindfulness priming into advertising copy or background music by guiding consumers to be more mindful. In addition, future research can explore more potential mediating variables. Although this research did not find the mediating effect of general emotion, specific emotions need to be studied, such as increased positivity and increased relaxation. Finally, because taste perception naturally has multisensory attributes [23], this research focuses on food products. Studies have pointed out that the consumption experience of service scenarios (e.g., dining in a restaurant, drinking coffee in a cafe) are also multisensory in nature [59]. Therefore, future research can extend to other products and fields.

## Figures and Tables

**Table 1:** Descriptive statistics and correlations of main variables in Study 2.

Variable	Mean	SD	1	2	3	4	5
Gender	1.48	0.50	-				
Age	28.37	5.24	0.14	-			
Mango preference	1.54	0.50	0.014	−0.079	-		
Number of sensory numbers	1.03	0.79	−0.033	−0.181	0.118	-	
Mindfulness	0.40	0.49	0.073	0.154	0.044	0.042	-
Purchase intentions	7.43	0.83	0.116	0.023	0.097	0.299 **	0.457 **

Note. *n* = 214. ** *p* < 0.01.

## Data Availability

The data are available from the corresponding author upon reasonable request.
